# Association Between High-Risk Human Papillomavirus Positivity and Ovarian Reserve Markers in Women with Unexplained Oligomenorrhea: A Retrospective Comparative Study

**DOI:** 10.3390/life16101642

**Published:** 2026-09-30

**Authors:** Ertugrul Sen

**Affiliations:** Gynecology and Obstetrics Department, Kayseri Eğitim ve Araştırma Hastanesi, Kayseri 38080, Turkey; drertugrulsen@gmail.com

**Keywords:** human papillomavirus, anti-Müllerian hormone, ovarian reserve, oligomenorrhea, antral follicle count

## Abstract

Aim: to evaluate the association between high-risk human papillomavirus (HR-HPV) positivity and ovarian reserve markers, particularly the anti-Müllerian hormone (AMH), in reproductive-age women presenting with unexplained oligomenorrhea. Methods: this single-center retrospective comparative study included women aged 25–35 years with unexplained oligomenorrhea, categorized into an HR-HPV-positive group (*n* = 257) and an HPV-negative control group (*n* = 1164). Ovarian reserve was assessed using serum AMH, basal follicle-stimulating hormone (FSH), luteinizing hormone (LH), estradiol (E2), and antral follicle count (AFC), with AMH measured irrespective of cycle day and other hormones on cycle days 2–5, while AFC was determined by transvaginal ultrasonography. Clinical and lifestyle variables were recorded, and multivariable logistic regression was used to identify factors independently associated with low AMH (<0.5 ng/mL). Results: the groups were comparable in age, body mass index, and parity. AMH was lower in HR-HPV-positive women than in controls (median 2.2 [IQR 1.0–3.8] vs. 2.7 [1.5–4.4] ng/mL; *p* < 0.001), as was AFC (10 [8–13] vs. 11 [9–14]; *p* < 0.001); the absolute differences were small (Cohen’s d ≤ 0.19). Low AMH was approximately twofold more frequent among HR-HPV-positive women (11.7% vs. 6.3%), and age (OR 1.36, 95% CI 1.26–1.47) and HR-HPV positivity (OR 2.07, 95% CI 1.24–3.47) were independently associated with low AMH; the association persisted at an alternative threshold of <1.1 ng/mL (adjusted OR 2.25, 95% CI 1.57–3.23). Conclusion: HR-HPV positivity was associated with lower AMH and AFC values in women with unexplained oligomenorrhea. These findings indicate a group-level association rather than a causal relationship and require confirmation in prospective studies incorporating HPV persistence and reproductive outcomes.

## 1. Introduction

Infertility affects approximately 8–12% of couples of reproductive age worldwide [1]. In female infertility, ovulatory disorders, tubal pathologies, endometrial factors, and sexually transmitted infections (STIs) play significant roles. The rate of infection-related female infertility has been reported to vary widely, ranging from 20% to 60% [2,3]. Human papillomavirus (HPV) is the most common sexually transmitted infection globally [4]. At least 40 HPV types are capable of infecting the anogenital epithelium. Based on their oncogenic potential, HPV types are classified as low risk (LR-HPV) or high risk (HR-HPV) [3,4]. Among these, HR-HPV types 16 and 18 are the primary oncogenic subtypes responsible for the majority of cervical cancer cases [4].

HPV is transmitted primarily through sexual contact, including penetrative intercourse and skin-to-skin genital contact. The estimated lifetime probability of acquiring HPV exceeds 80% among sexually active women [5]. Established risk factors for HPV acquisition include early sexual debut, a higher number of lifetime sexual partners, the sexual behavior of the male partner, smoking, oral contraceptive use, immunosuppression, and coexisting sexually transmitted infections [6]. Most cervical HPV infections are transient and cleared spontaneously within approximately two years, whereas persistent infection with high-risk types is the key determinant of progression to cervical intraepithelial neoplasia and cervical cancer [7].

Systematic reviews and meta-analyses investigating the relationship between HPV and infertility have reported that HR-HPV infections are more prevalent among infertile women compared with the general population, suggesting that HR-HPV may represent a potential risk factor for female infertility [3]. However, a large-scale cohort study failed to demonstrate an independent causal relationship between HR-HPV positivity or persistent infection and the subsequent development of infertility. Therefore, HR-HPV has been proposed to act as a co-factor interacting with other infections and risk determinants, rather than as a sole independent etiologic factor [8].

More recently, experimental and clinical studies have indicated that HPV may affect ovarian reserve through mechanisms involving oxidative stress, chronic inflammation, and granulosa cell dysfunction [9,10]. HPV, particularly through its early proteins, has been shown to interact with the mitochondrial membrane, increase the production of reactive oxygen species, suppress antioxidant defense systems, and induce DNA damage. These processes may contribute to gamete dysfunction and loss of fertility in both sexes [10,11]. The anti-Müllerian hormone (AMH), the most widely used biochemical marker for evaluating ovarian reserve, reflects the number of small antral follicles and provides sensitive insights into the follicular pool [12].

The number of studies directly examining the relationship between HPV and ovarian reserve is limited. A case–control study reported that AMH levels were lowest in the CIN III group, and that the frequency of “diminished ovarian reserve” was significantly higher among HPV-positive women [13].

These findings suggest that the association between HPV and female infertility may not be limited to cervical and tubal factors, but may also potentially involve the ovarian reserve. The aim of this study is to investigate the relationship between HR-HPV infection and ovarian reserve indicators [AMH, baseline follicle-stimulating hormone (FSH), luteinizing hormone (LH), estradiol (E2), and antral follicle count (AFC)] in women.

## 2. Method

**Figure 1:**
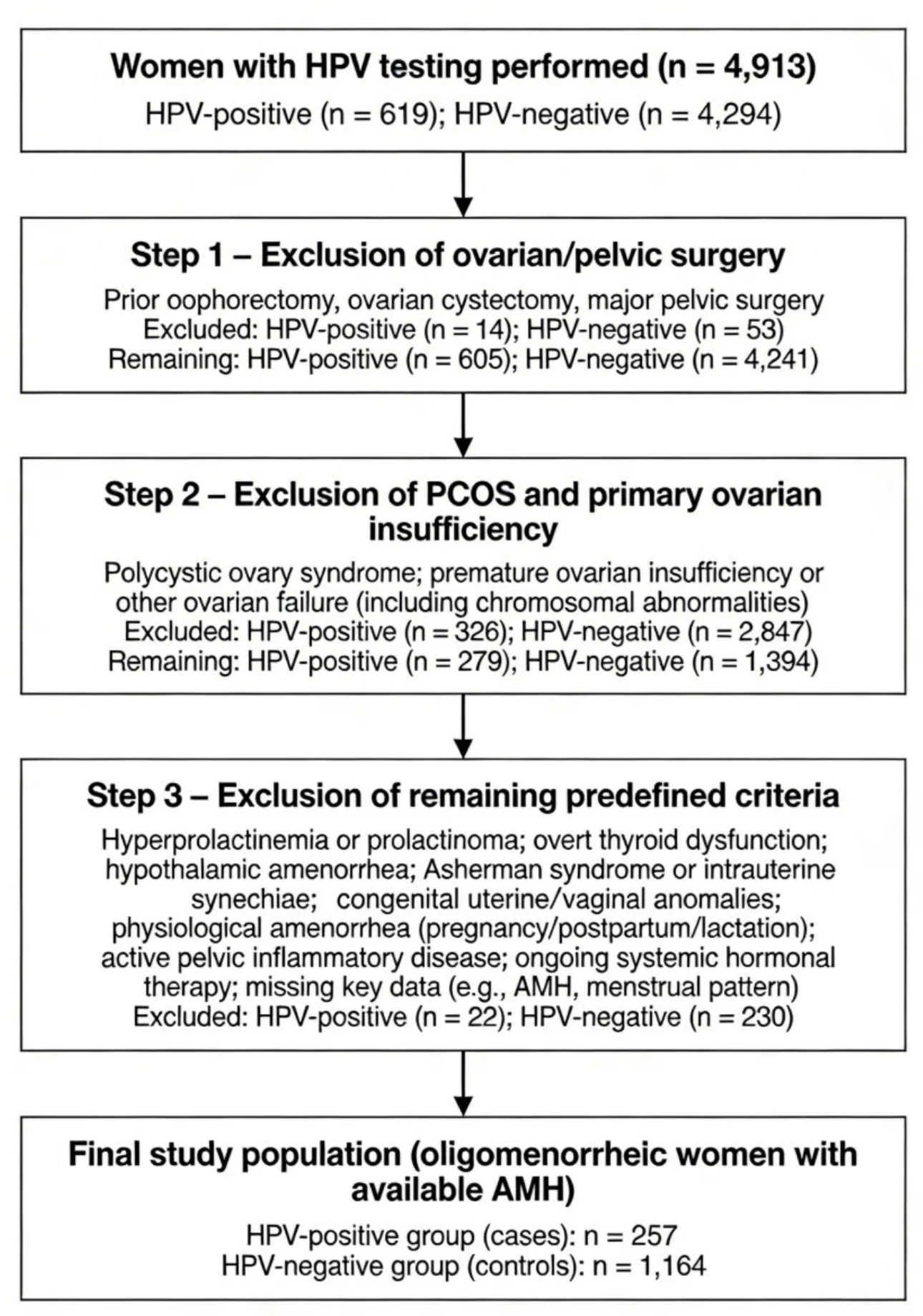
Flow diagram of participant selection in this retrospective comparative study.

This study was designed as a single-center retrospective comparative study conducted on women who presented to the outpatient clinic of the Department of Obstetrics and Gynecology at Kayseri City Training and Research Hospital. The study was conducted between January 2019 and December 2022. Rather than consecutive enrollment, all patients who met the predefined eligibility criteria during the study period were identified from the institutional records and included in the relevant study group according to their HPV status. The inclusion criteria for the case group were as follows: being between 25–35 years of age, having documented HPV positivity, and having a menstrual cycle pattern characterized by unexplained oligomenorrhea. The control group consisted of HPV-negative patients who presented with unexplained oligomenorrhea. The exclusion criteria included a history of oophorectomy, ovarian cystectomy, or major pelvic surgery; having received chemotherapy or pelvic radiotherapy; the presence of polycystic ovary syndrome; a diagnosis consistent with premature ovarian insufficiency or chromosomal anomalies; uncontrolled endocrinopathies; active pelvic inflammatory disease; and ongoing systemic hormonal therapy. In this study, patients with cycle disorders due to hyperprolactinemia or prolactinoma, overt thyroid dysfunction, hypothalamic amenorrhea (anorexia, significant weight loss, intense exercise, severe stress), pituitary adenoma or other pituitary masses, Asherman syndrome or intrauterine synechiae detected after major intrauterine surgery, congenital uterine/vaginal anomalies, physiological amenorrhea related to pregnancy, postpartum or lactation, a history of gonadotoxic chemotherapy/radiotherapy, and those receiving hormonal contraceptives or GnRH analogue/progestin therapy that markedly alter menstrual patterns were excluded. Unexplained oligomenorrhea was defined as an infrequent ovulatory/anovulatory bleeding pattern in a woman of reproductive age, characterized by a menstrual cycle interval longer than 35 days or the presence of fewer than nine cycles per year, in the absence of pregnancy, lactation, perimenopause, overt endocrine (for example, hyperprolactinemia, thyroid dysfunction), structural (for example, Asherman syndrome) or systemic causes [14]. Of 4913 women who underwent HPV testing during the study period (619 HPV-positive and 4294 HPV-negative), 67 were excluded for prior oophorectomy, ovarian cystectomy, or major pelvic surgery; 3173 for polycystic ovary syndrome, premature ovarian insufficiency, or other ovarian failure; and 252 for the remaining predefined exclusion criteria, including missing key data (e.g., AMH or menstrual pattern documentation), leaving 1421 women for analysis (257 HR-HPV-positive and 1164 HPV-negative). Patient selection is illustrated in Figure 1 as a flow chart. The study received ethical approval from the University Clinical Research Ethics Committee (Decision no: 2022/787; date: 7 December 2022). All procedures were performed in accordance with the ethical standards of the institutional and/or national research committee and in compliance with the 1964 Helsinki Declaration and its subsequent revisions or similar ethical guidelines. Owing to the retrospective design of the study, the requirement for written informed consent was waived by the ethics committee.

The restriction to women with unexplained oligomenorrhea was intended to create a clinically comparable cohort and reduce heterogeneity related to known causes of menstrual dysfunction. However, this selection limits the generalizability of the findings to healthy women, women with regular cycles, and infertile populations overall.

HPV DNA testing was performed on cervical samples using the cobas HPV test on the cobas 6800 system (Roche Molecular Systems, Branchburg, NJ, USA), a fully automated real-time PCR assay that individually identifies HPV16 and HPV18 and reports twelve other high-risk genotypes (31, 33, 35, 39, 45, 51, 52, 56, 58, 59, 66, 68) as a pooled result, consistent with the genotypes classified as carcinogenic or probably carcinogenic by the International Agency for Research on Cancer (IARC) [4]. Cervical cytology results, when available, were recorded and classified according to the Bethesda system; cytology was available only for the HPV-positive group.

To assess ovarian reserve, AMH, basal FSH, LH, E2, and AFC parameters were used [12,13]. A single venous blood sample was obtained from all participants irrespective of cycle day [13]. Serum AMH was measured by electrochemiluminescence immunoassay (ECLIA) using the Elecsys AMH assay on the cobas e801 analyzer (Roche Diagnostics, Mannheim, Germany), with a measuring range of 0.010–23 ng/mL and intra- and inter-assay coefficients of variation of 1.8% and 2.7%, respectively. Serum FSH, LH, and E2 levels were measured in samples collected in the morning on days 2–5 of the menstrual cycle [12]. Transvaginal ultrasonography was performed on cycle days 2–5 using a Philips ClearVue 550 ultrasound system (Philips Ultrasound, Bothell, WA, USA) equipped with a transvaginal transducer, and all antral follicles measuring 2–10 mm in both ovaries were counted to determine the total AFC. AFC was assessed by a single gynecologist with at least five years of experience in gynecological ultrasonography, who was not blinded to HPV status. Antral follicle counts between 0–7 were classified as low, those between 8–24 as normal, and those greater than 24 as PCO [15,16]. When AMH values were classified according to age, the 5th–95th percentile range for women aged 25–35 years was defined as 0.5–7.0 ng/mL [17,18]. Values below this level were defined as low AMH. For the present analysis, AMH < 0.5 ng/mL was classified as low AMH based on the cited age-specific reference framework. This threshold was used as an operational definition for the regression analysis and should not be interpreted as a universally validated diminished-ovarian-reserve cutoff for all women aged 25–35 years. Patients with missing data for the variables required for the analysis were excluded from the study. In cases of duplicate records, only the first recorded AMH measurement was retained for analysis.

Clinical variables such as age, body mass index (BMI), smoking status, alcohol consumption, parity, and HPV vaccination status (at least one dose) were recorded. Smoking was defined as daily tobacco use regardless of quantity, and alcohol consumption as intake at least once per week regardless of amount; because consumption quantities were not systematically documented, both were modeled as binary variables. Patients with a BMI ≤ 18.4 were classified as underweight, those with a BMI of 18.5–24.9 as normal, 25.0–29.9 as overweight, and ≥30 as obese [19].

### Statistical Analysis

Continuous variables were tested for normality using the Shapiro–Wilk test. Because all continuous variables show non-normal distributions, they are presented as median (interquartile range, IQR) together with mean ± standard deviation, and were compared using the Mann–Whitney U test. Categorical variables were compared using the chi-square test or Fisher’s exact test, as appropriate. Mean differences with 95% confidence intervals (CI) and standardized effect sizes (Cohen’s d) were calculated for the primary ovarian reserve markers. AMH and AFC were prespecified as the primary outcomes; the remaining comparisons were considered descriptive, and no adjustment for multiple comparisons was applied to these secondary analyses. Logistic regression analysis was used to examine the association between HPV status and the likelihood of low AMH, expressed as odds ratios (OR) with 95% CIs. The dependent variable was coded as 1 for low AMH (<0.5 ng/mL) and 0 otherwise. Covariates were selected a priori based on clinical relevance and published evidence regarding potential associations with ovarian reserve and HPV status; stepwise selection was not used. The final multivariable model included age, smoking, alcohol consumption, HPV vaccination, BMI, HR-HPV positivity, LSIL, and HSIL. The model included 103 low-AMH events and eight covariates (events per variable = 12.9), and multicollinearity was assessed using variance inflation factors (all ≤1.24). A sensitivity analysis was performed using an alternative low-AMH threshold of <1.1 ng/mL. Data recording and statistical analyses were performed using SPSS software (version 17, SPSS Inc., Chicago, IL, USA), and all analyses were independently verified. A two-sided *p* value of <0.05 was considered to indicate statistical significance.

## 3. Results

Among 1421 women included in the study, 257 were HPV-positive and 1164 were controls. The two groups were comparable in terms of age and body mass index, both as continuous variables and across BMI categories, as well as parity distribution (Table 1). Smoking and alcohol consumption tended to be more frequent in the HPV-positive group, although these differences did not reach statistical significance. HPV vaccination was significantly more common in the HPV-positive group compared with controls (21.8% vs. 14.8%; *p* = 0.006). Regarding ovarian reserve markers, both AFC and AMH were lower in HPV-positive women than in controls. Median AFC was 10.0 (IQR 8.0–13.0) in HPV-positive women versus 11.0 (IQR 9.0–14.0) in controls (mean 11.4 ± 5.9 vs. 12.3 ± 5.5; mean difference −0.94, 95% CI −1.70 to −0.19; Cohen’s d = −0.17; *p* < 0.001). Median AMH was 2.2 ng/mL (IQR 1.0–3.8) versus 2.7 ng/mL (IQR 1.5–4.4) (mean 2.8 ± 2.8 vs. 3.4 ± 2.8 ng/mL; mean difference −0.53, 95% CI −0.91 to −0.15; Cohen’s d = −0.19; *p* < 0.001). When analyzed categorically, AFC categories did not differ significantly between groups (*p* = 0.062), whereas low AMH (<0.5 ng/mL) was significantly more frequent in HPV-positive women (11.7% vs. 6.3%; risk difference 5.4%, 95% CI 1.2–9.6%; OR 1.98, 95% CI 1.26–3.09; *p* = 0.004). In a sensitivity analysis using an alternative threshold of <1.1 ng/mL, AMH below the threshold was likewise more frequent in HPV-positive women (30.0% vs. 16.8%; adjusted OR 2.25, 95% CI 1.57–3.23; *p* < 0.001). Follicle-stimulating hormone (FSH), luteinizing hormone (LH), and estradiol (E2) levels did not differ significantly between the groups.

Among HPV-positive women, HPV16 was detected in 81 patients (31.5%), HPV18 in 22 patients (8.6%), and other high-risk HPV types in 173 patients (67.3%); percentages exceed 100% because 26 women (10.1%) had multiple high-risk types (25 with two types and one with three types). Genotype information was not documented in 8 women. In an exploratory analysis, AMH levels did not differ among women positive for HPV16 only (n = 61; median 2.0 ng/mL), HPV18 only (n = 14; median 2.2 ng/mL), other high-risk types only (n = 148; median 2.2 ng/mL), and multiple types (n = 26; median 1.4 ng/mL) (Kruskal–Wallis *p* = 0.89); this analysis was underpowered, particularly for the HPV18 subgroup, and should be interpreted with caution. In terms of cytological findings in the HPV-positive group, 144 women (56.0%) had atypical squamous cells of undetermined significance (ASC-US), 42 (16.3%) had low-grade squamous intraepithelial lesions (LSIL), 16 (6.2%) had high-grade squamous intraepithelial lesions (HSIL), and 55 (21.4%) had normal cytology; one woman with concomitant LSIL and HSIL findings was classified according to the more severe result. Cervical cytology was not available for the control group. Within the HPV-positive group, AMH levels did not differ significantly between women with HSIL and those with normal cytology (2.6 ± 3.8 vs. 3.1 ± 3.1 ng/mL; *p* = 0.262), and low AMH was observed in 3 of 16 (18.8%) women with HSIL versus 7 of 55 (12.7%) with normal cytology (*p* = 0.68).

Factors associated with low AMH (<0.5 ng/mL) were evaluated using univariate and multivariable logistic regression (Table 2). In the multivariable model, age (OR = 1.36, 95% CI 1.26–1.47; *p* < 0.001) and HR-HPV positivity (OR = 2.07, 95% CI 1.24–3.47; *p* = 0.006) were independently associated with low AMH.

## 4. Discussion

In this study, the relationship between HR-HPV infection and ovarian reserve markers in women was investigated. It was determined that the mean AMH level was lower in the case group of HPV-positive women aged 25–35 years who presented with complaints of oligomenorrhea. When factors that could predict low AMH levels were examined, age and HPV positivity were independently associated with low AMH. This finding is generally consistent with the results of systematic reviews and meta-analyses indicating that HR-HPV infection may represent a potential risk factor for female infertility [3,8]. However, a substantial portion of the current literature has focused primarily on infertility prevalence, pregnancy rates, and assisted reproductive technology (ART) outcomes rather than directly assessing ovarian reserve [3,8].

Age-specific AMH nomogram studies have shown that serum AMH levels peak in the early twenties, then gradually decline toward the thirties, with a more pronounced decrease emerging in the late thirties to forties. In reference range studies, although median AMH values at 26–30 years are lower than those at 20–25 years, they remain comparable to those at 31–35 years, and show a marked decline only from 36 years onward; this supports the notion that the 25–35-year interval represents a window in which a “physiological, predictable decline” occurs, but the impact of advanced age has not yet become prominent [20,21,22,23]. Reviews summarizing ovarian ageing and ovarian reserve tests emphasize that optimal fertility is observed between 20 and 30 years of age, whereas a marked and accelerated decline in both oocyte quantity and quality occurs after the age of 35 [24,25]. To limit the strong effect of age-related loss of ovarian reserve, only women aged 25–35 years were included in our study.

A considerable proportion of studies evaluating the association between HPV infection and female infertility have shown that HR-HPV positivity is more frequent in infertile populations than in the general population [3,8]. However, in a large population-based cohort study with long-term follow-up data, no independent causal relationship could be demonstrated between HR-HPV positivity, or even persistent infection with the same type, and the subsequent development of infertility [8]. In that study, after adjustment for confounding variables such as age, smoking, educational level, previous pelvic inflammation, and Chlamydia trachomatis infection, HR-HPV positivity was reported not to significantly increase the risk of infertility [8]. Age, which is the strongest determinant of ovarian reserve, as well as smoking, body mass index (BMI), and alcohol consumption are important factors [2,3,8]. In our study, no significant differences were observed between the groups with respect to these confounding factors.

Rocha et al. evaluated the presence of HR-HPV DNA by performing multiple sampling from both the lower and upper genital tract (cervix, vagina, endometrium, tubal lavage, peritoneal fluid, ovarian biopsy) during laparoscopy, and demonstrated that HR-HPV positivity in the upper genital tract was significantly more frequent in infertile women and those with endometriosis. This finding suggests that an HPV infection originating in the cervix may ascend to the uterine cavity, fallopian tubes, and peritoneal surfaces, thereby creating a chronic inflammatory microenvironment at these sites [9].

The following mechanisms remain hypothetical, as no inflammatory, oxidative, or molecular biomarkers were measured in the present study. Experimental studies have reported that early HR-HPV proteins (particularly E2, E6, and E7) interact with mitochondria, increase reactive oxygen species production, impair antioxidant defenses, and induce DNA damage [10], processes that could, in principle, disrupt granulosa cell function, steroidogenesis, and the follicular microenvironment [11,26]. In a case–control study conducted by Fakor et al. on 219 women aged 25–35 years, AMH levels were significantly lower in HR-HPV-positive women than in HPV-negative women, and the CIN III subgroup had the lowest AMH levels; the higher frequency of diminished ovarian reserve among HR-HPV-positive women supported a potential link between chronic HPV infection and ovarian reserve [13]. In our study, HR-HPV positivity was independently associated with lower AMH levels, the most widely used marker of ovarian reserve; however, the retrospective design does not allow any causal inference.

The clinical significance of these differences warrants careful interpretation. The standardized effect sizes were small (Cohen’s d = 0.19 for AMH and 0.17 for AFC), and absolute differences of this magnitude would not, by themselves, alter the fertility management of an individual patient; with more than 1400 participants, even small differences reach statistical significance. The shift in the lower tail of the AMH distribution appears more relevant: the proportion of women with low AMH was approximately twofold higher among HPV-positive women (11.7% vs. 6.3%; risk difference 5.4%, 95% CI 1.2–9.6%), and this association persisted when the alternative threshold of <1.1 ng/mL was applied. These findings represent a group-level association and do not constitute a basis for individual-level screening or prognostication.

The relationship between CIN grade and AMH suggests the possibility of a “dose–response” effect related to factors such as duration of HPV exposure, viral load, and persistence. CIN III represents a more advanced stage in terms of both viral persistence and the accumulation of genetic/epigenetic damage in the host cells [4]. In the study by Fakor et al., AMH levels were found to be significantly lower in CIN III cases compared with women with normal cervical histology, and the proportion of diminished ovarian reserve was highest in this group, suggesting that longer and more intense exposure to HPV may lead to cumulative damage to ovarian reserve [13]. In our study, AMH levels in patients with HSIL lesions did not differ significantly compared with those in patients without cervical cytological abnormalities. This may be due to the insufficient number of HSIL cases in our study and the fact that patients were generally selected on the basis of a specific indication such as unexplained oligomenorrhea. Contrary to the studies reported in the literature, another possible explanation for our findings is that the impact on ovarian reserve may be determined by more complex factors—such as systemic inflammatory responses, concomitant infections, or host genetic–immune response characteristics—rather than by the burden of cervical lesions per se [8,11,13].

It has been shown that in women with HR-HPV positivity undergoing assisted reproductive technologies, clinical pregnancy rates are lower, while rates of early pregnancy loss and possibly preterm birth are higher. At the same time, the presence of seminal HPV has been reported to impair sperm DNA integrity, thereby adversely affecting fertilization and embryo development, which in turn reduces treatment success at the couple level [11].

An unexpected finding was the higher rate of HPV vaccination among HPV-positive women (21.8% vs. 14.8%). Several non-causal explanations are plausible. First, the timing of vaccination relative to HPV testing was not recorded, and some women may have received a catch-up vaccination after a positive HPV test or after the onset of sexual activity, when exposure may already have occurred. Second, prophylactic HPV vaccines do not accelerate the clearance of established infections [27]. Third, because HPV vaccination is not included in the national immunization program in Turkey and is paid for out of pocket, vaccinated women may represent a group with greater health-care engagement and, consequently, a higher probability of undergoing HPV testing, a form of detection bias. Finally, 67.3% of HPV-positive women carried non-16/18 high-risk types, which are not covered by vaccines targeting HPV16/18. Notably, vaccination status was not associated with low AMH in the multivariable model (OR 1.00, 95% CI 0.57–1.73), indicating that vaccination did not confound the main association.

Although definitive clinical recommendations are beyond the scope of this observational study, several guideline-based principles apply to HPV-positive women who plan pregnancy. HPV positivity alone is not a reason to postpone conception, as most infections clear spontaneously within approximately two years [7]. Cervical lesions should be managed according to current guidelines, and unnecessary excisional procedures should be avoided in women who desire fertility, given the association of excisional treatment with preterm birth [28]. Vaccination may be offered to unvaccinated women, with counseling that it does not treat established infection [27]. Finally, smoking cessation should be encouraged, as smoking has been associated with delayed HPV clearance [29] and is an established modifiable risk factor for diminished ovarian reserve.

This study has several limitations. Its retrospective, single-center design precludes determination of temporality, and reverse causation cannot be excluded; women with diminished ovarian reserve may be more likely to undergo gynecological evaluation, including HPV testing, particularly given that women younger than 30 years fall outside the national HPV screening program and are therefore tested on the basis of clinical indication. The restriction to women aged 25–35 years with unexplained oligomenorrhea limits the generalizability of the findings to healthy women, women with regular cycles, and infertile populations overall. Important confounders—including prior sexually transmitted infections and Chlamydia trachomatis, sexual behavior, socioeconomic characteristics, contraceptive history, infertility duration, metabolic factors, vitamin D status, and autoimmune disease—were unavailable, and residual confounding is therefore likely; HPV positivity correlates strongly with sexual behavior and STI exposure, and the observed association may partly reflect these unmeasured factors. HPV status was based on a single cervical test; persistence, viral load, duration of infection, and upper genital tract HPV status were not assessed, so transient infection cannot be distinguished from persistent infection, which substantially restricts biological interpretation and precludes the evaluation of a dose–response relationship. Genotype information was undocumented in eight HPV-positive women, and cervical cytology was not available for the control group. AFC was measured by an operator who was not blinded to HPV status, and interobserver variability could not be assessed. Although the primary comparisons remained significant after correction for multiple testing, the absolute differences were small, secondary analyses were exploratory, and concomitant genital infections could not be completely excluded; no inflammatory or oxidative stress biomarkers were measured. One of the strengths of the study is that ovarian reserve was evaluated using both biochemical (AMH, FSH, LH, E2) and ultrasonographic (AFC) parameters in a large, well-defined cohort. In addition, the absence of significant differences in age, BMI, smoking, and alcohol consumption between the groups reduces confounding by these measured factors.

In conclusion, HR-HPV positivity was associated with lower AMH and AFC values and a higher frequency of low AMH in women aged 25–35 years with unexplained oligomenorrhea. These findings indicate an association rather than a causal relationship. Whether they have clinical implications for fertility assessment should be determined in prospective studies incorporating HPV persistence, pregnancy, and assisted reproduction outcomes. Current evidence indicates a multifaceted relationship between HPV infection and female reproductive health, and the potential relevance of HPV to ovarian reserve is only beginning to be elucidated.

## Figures and Tables

**Table 1:** Clinical and demographic characteristics of HPV-positive and control groups.

	HPV + (n = 257)	Control (n = 1164)	Effect Size (95% CI)	*p*
**Age a**	29.9 ± 2.9 30 (28–32)	29.6 ± 3.0 29 (27–32)	MD 0.25 (−0.16 to 0.66)	0.159
**Body Mass Index a (kg/m^2^)**	25.1 ± 5.6 23 (21–28)	24.9 ± 5.5 23 (21–28)	MD 0.11 (−0.63 to 0.86)	0.752
**Body Mass Index b (kg/m^2^)**			—	0.992
**Underweight (≤18.4)**	4 (1.6)	18 (1.5)		
**Normal weight (18.5–24.9)**	166 (64.6)	762 (65.5)		
**Overweight (25.0–29.9)**	52 (20.2)	233 (20.0)		
**Obesity (≥30)**	35 (13.6)	151 (13.0)		
**Parity a**	1.2 ± 1.1 1 (0–2)	1.3 ± 1.2 1 (0–2)	MD −0.11 (−0.28 to 0.05)	0.254
**Parity b**			—	0.312
**Nulliparous**	84 (32.7)	368 (31.6)		
**Uniparous**	68 (26.5)	259 (22.3)		
**Multiparous**	93 (36.2)	460 (39.5)		
**Unknown**	12 (4.7)	77 (6.6)		
**Smoking b**	99 (38.5)	390 (33.5)	OR 1.24 (0.94–1.64)	0.126
**Alcohol consumption b**	77 (30.0)	290 (24.9)	OR 1.29 (0.96–1.74)	0.094
**HPV vaccination b**	56 (21.8)	172 (14.8)	OR 1.61 (1.15–2.25)	0.006
**Follicle-stimulating hormone a**	7.0 ± 3.3 6.3 (5.3–7.7)	6.7 ± 3.1 6.4 (5.3–7.6)	MD 0.29 (−0.14 to 0.72)	0.959
**Luteinizing hormone a**	6.0 ± 3.6 5.3 (3.7–6.9)	6.2 ± 4.9 5.3 (4.0–7.1)	MD −0.28 (−0.91 to 0.36)	0.481
**Estradiol a**	56.2 ± 64.5 47.0 (32.0–63.4)	51.3 ± 34.1 45.5 (33.7–61.3)	MD 4.9 (−0.7 to 10.5)	0.978
**Antral follicle count a**	11.4 ± 5.9 10 (8–13)	12.3 ± 5.5 11 (9–14)	MD −0.94 (−1.70 to −0.19) d = −0.17	<0.001
**Antral follicle count b**			—	0.062
**Low (0–7)**	37 (14.4)	110 (9.5)		
**Normal (8–24)**	209 (81.3)	1001 (86.0)		
**PCO (>24)**	11 (4.3)	53 (4.6)		
**Anti-Müllerian hormone a (ng/mL)**	2.8 ± 2.8 2.2 (1.0–3.8)	3.4 ± 2.8 2.7 (1.5–4.4)	MD −0.53 (−0.91 to −0.15) d = −0.19	<0.001
**Anti-Müllerian hormone b (ng/mL)**			Low AMH: RD 5.4% (1.2 to 9.6) OR 1.98 (1.26–3.09)	0.010
**Low (<0.5)**	30 (11.7)	73 (6.3)		
**Normal (0.5–7.0)**	205 (79.8)	982 (84.4)		
**High (>7.0)**	22 (8.6)	109 (9.4)		
**Cervical cytology b (HPV-positive group only)**		Not available	—	—
**ASC-US**	144 (56.0)			
**LSIL**	42 (16.3)			
**HSIL**	16 (6.2)			
**Normal**	55 (21.4)			

a: mean ± standard deviation and median (interquartile range); compared with the Mann–Whitney U test. b: n (%); compared with the chi-square test. MD = mean difference; RD = risk difference; OR = odds ratio; d = Cohen’s d effect size. Six control participants with AMH values of 7.02–7.08 ng/mL, previously unclassified, were assigned to the high AMH category. Cervical cytology was available only for the HPV-positive group; one woman with concomitant LSIL and HSIL was classified by the more severe finding. HPV: human papillomavirus; AMH: anti-Müllerian hormone; ASC-US: atypical squamous cells of undetermined significance; LSIL: low-grade squamous intraepithelial lesion; HSIL: high-grade squamous intraepithelial lesion; PCO: polycystic ovary pattern.

**Table 2:** Univariate and multivariable logistic regression analyses of factors associated with low anti-Müllerian hormone levels (<0.5 ng/mL).

	Univariate			Multivariate		
	OR	95% CI	*p*	OR	95% CI	*p*
Age, per 1-year increase	1.35	1.25–1.46	<0.001	1.36	1.26–1.47	<0.001
Smoking (ref: non-smoker)	0.98	0.64–1.50	0.924	0.88	0.56–1.37	0.557
Alcohol consumption (ref: none)	1.08	0.69–1.69	0.744	0.99	0.61–1.59	0.957
HPV vaccination (ref: unvaccinated)	1.12	0.66–1.90	0.681	1.00	0.57–1.73	0.989
BMI, per 1 kg/m^2^ increase	0.97	0.93–1.01	0.179	0.97	0.93–1.01	0.189
HR-HPV positivity (ref: HPV-negative)	1.98	1.26–3.09	0.003	2.07	1.24–3.47	0.006
LSIL (ref: normal cytology)	1.33	0.46–3.78	0.599	0.70	0.22–2.20	0.537
HSIL (ref: normal cytology)	3.01	0.84–10.74	0.089	1.58	0.40–6.18	0.510

OR: Odds ratio, CI: Confidence interval. BMI: body mass index; HPV: human papillomavirus; LSIL: low-grade squamous intraepithelial lesion; HSIL: high-grade squamous intraepithelial lesion. Outcome coded 1 = low AMH (<0.5 ng/mL), 0 = otherwise (103 events). Covariates were selected a priori; stepwise selection was not used. Events per variable = 12.9; variance inflation factors all ≤1.24.

## Data Availability

The datasets generated and analyzed during our study are not publicly available due to patient confidentiality and data protection regulations. However, they can be obtained from the corresponding author upon reasonable request.
